# Data on occurrence of miRNA precursors in the *Cucurbita maxima* phloem sap

**DOI:** 10.1016/j.dib.2019.105083

**Published:** 2020-01-03

**Authors:** Eugeny A. Tolstyko, Alexander A. Lezzhov, Anna D. Solovieva, Andrey G. Solovyev

**Affiliations:** a Department of Virology, Biological Faculty, Moscow State University, Moscow 119234, Russia; b Faculty of Bioengineering and Bioinformatics, Moscow State University, Moscow 119991, Russia; c Belozersky Institute of Physico-Chemical Biology, Moscow State University, Moscow 119992, Russia; d Sechenov First Moscow State Medical University, Institute of Molecular Medicine, Moscow 119991, Russia

**Keywords:** miRNA, pri-miRNA, miRNA precursor, Phloem, Phloem RNA

## Abstract

The phloem sieve elements (SEs), enucleate cells, contain RNAs, which are imported from surrounding tissues and cells, mostly companion cells tightly associated with SEs, and transported via the phloem over the whole plant body. The RNA phloem transport is essential for plant individual development and responses to environmental cues. Recently, we identified primary miRNA (pri-miRNA) sequences in *de novo* assembled transcriptome of *Cucurbita maxima* phloem sap and reported 11 most abundant pri-miRNAs [1]. Here, we provide the output of this analysis in complete detail. For the full set of pri-miRNAs identified in the *C. maxima* phloem sap transcriptome, data on relative abundance are provided along with annotated sequence data.

**Table:** Specifications Table

Subject	Plant Science
Specific subject area	miRNA-based regulation of gene expression in plants
Type of data	Table
How data were acquired	Phloem transcriptome was assembled by SPAdes 3.12.0. Contigs containing miRNA precursors were identified by using BLAST. Alignments of primary reads with contigs were performed using Bowtie2.
Data format	Raw data, analyzed data
Parameters for data collection	Database search, dataset selection.
Description of data collection	Transcriptome sequencing data for *C. maxima* phloem sap were retrieved from NCBI Sequence Read Archive.
Data source location	The dataset is provided in Supplementary Fig. S1
Data accessibility	With the article; raw data are provided in Supplementary Fig. S1, analyzed data are presented in Table 1
Related research article	E. A. Tolstyko, A. A. Lezzhov, and A. G. Solovyev. Identification of miRNA precursors in the phloem of Cucurbita maxima. PeerJ, 7 (2019) e8269. https://doi.org/10.7717/peerj.8269.

**Table:** 

**Value of the Data** • This is the first report on the set of pri-miRNAs that are present in the phloem sap. • The reported data can be valuable for scientists studying cell-to-cell and phloem transport of RNA and, in general, molecular mechanisms of signalling in plants. • These data will be useful for rational, knowledge-based selection of model pri-miRNAs for experimental analyses of molecular mechanism of pri-miRNA transport into SEs and long-distance trafficking via the phloem, as well as studies of phloem pri-miRNAs roles in regulation of gene expression.

## 1 Data description

Sequence analysis of transcriptome sequencing data for *Cucurbita maxima* phloem sap revealed the presence of 35 contigs showing significant (e-value less than 1e-15) sequence similarity to known pri-miRNAs of *Cucumis melo* [1]; raw data on sequences of identified contigs are presented in Supplementary Fig. S1. In a further analysis of the raw data, a relative abundance of each of these contigs in the phloem sap was characterized by the number of primary reads that could be aligned to this contig, read count normalized per 100 nucleotides of contig sequence, and average coverage (Table 1).

**Table 1:** Identification of pri-miRNA sequences in the *Cucurbita maxima* phloem sap.

*C. melo* pre-miRNA	*C. maxima* Contig name	Alignment length	E value	Contig length	Coverage	Reads aligned	Reads per 100 nucl.
cme-MIR156d	P69980	56	1.E−17	174	2.78	9	5.17
cme-MIR156g	P18642	102	2.E−34	880	5.55	100	11.36
cme-MIR159a	P17547	209	7.E−51	925	22.13	433	46.81
cme-MIR159a	P21134	209	2.E−65	784	72.92	1216	155.1
cme-MIR160a	P48351	86	1.E−30	261	5.19	26	9.96
cme-MIR160b	P25386	90	1.E−30	639	41.21	479	74.96
cme-MIR160b	P29894	90	1.E−30	515	29.6	184	35.73
cme-MIR162	P50997	99	6.E−41	245	1.89	8	3.27
cme-MIR162	P60916	88	2.E−28	200	3.5	12	6
cme-MIR166b	P44166	103	1.E−36	293	6.56	38	12.97
cme-MIR166d	P19669	120	1.E−25	841	98.02	1795	213.44
cme-MIR166e	P31502	287	3.E−32	479	20.68	200	41.75
cme-MIR167c	P88391	103	1.E−24	146	3.8	8	5.48
cme-MIR167e	P45184	117	4.E−19	285	1.59	9	3.16
cme-MIR167f	P25784	127	9.E−20	627	8.74	111	17.7
cme-MIR168	P7902	218	2.E−52	1508	10.91	351	23.28
cme-MIR169g	P48844	120	2.E−23	258	4.75	22	8.53
cme-MIR169n	P12201	148	6.E−17	1201	7.95	199	16.57
cme-MIR169n	P84102	54	2.E−16	153	3.01	8	5.23
cme-MIR169r	P49298	91	8.E−20	255	5.34	27	10.59
cme-MIR171i	P3867	187	8.E−43	1986	47.98	2306	116.11
cme-MIR171i	P5314	192	2.E−26	1784	11.3	466	26.12
cme-MIR172b	P53263	84	6.E−16	232	7.84	32	13.79
cme-MIR2111a	P53142	55	6.E−16	233	3.28	16	6.87
cme-MIR319a	P20012	188	2.E−70	828	42.88	748	90.34
cme-MIR319b	P89332	62	4.E−22	144	3.83	8	5.56
cme-MIR319c	P53520	87	4.E−30	231	4.39	18	7.79
cme-MIR319d	P20793	192	1.E−53	797	20.76	333	41.78
cme-MIR390a	P37982	116	5.E−24	364	26.74	236	64.84
cme-MIR390b	P38121	135	3.E−21	362	18.86	132	36.46
cme-MIR393a	P10713	113	8.E−28	1292	102.9	2777	214.94
cme-MIR396b	P29987	149	5.E−32	513	22.81	235	45.81
cme-MIR530b	P55325	109	4.E−24	223	1.64	6	2.69
cme-MIR854	P7676	106	6.E−32	1528	108.11	3640	238.22
cme-MIR854	P8823	135	5.E−33	1428	59.27	1792	125.49

Sequences showing significant (e-value less than 1e-15) sequence similarity to the following *C. melo* miRNA precursors were not found in *C. maxima* phloem sap transcriptome: miR156a, miR156b, miR156c, miR156e, miR156f, miR156h, miR156i, miR156j, miR159b, miR160c, miR160d, miR164a, miR164b, miR164c, miR164d, miR166a, miR166c, miR166g, miR166h, miR166i, miR167a, miR167b, miR167d, miR169a, miR169b, miR169c, miR169d, miR169e, miR169f, miR169h, miR169i, miR169j, miR169k, miR169l, miR169m, miR169o, miR169p, miR169q, miR169s, miR169t, miR171a, miR171b, miR171c, miR171d, miR171e, miR171f, miR171g, miR171h, miR172a, miR172c, miR172d, miR172e, miR172f, miR1863, miR2111b, miR390c, miR390d, miR393b, miR393c, miR394a, miR394b, miR395a, miR395b, miR395c, miR395d, miR395e, miR395f, miR396a, miR396c, miR396d, miR396e, miR397, miR398a, miR398b, miR399a, miR399b, miR399c, miR399d, miR399e, miR399f, miR399g, miR408, miR477a, miR477b, miR530a, miR7129, miR7130, miR828, miR845, miR858.

## 2 Experimental design, materials, and methods

Transcriptome sequencing data for *C. maxima* phloem sap (SRX146322) were downloaded using fastq-dump tool of NCBI SRA Toolkit 2.9.0. (http://ncbi.github.io/sra-tools/). Reads quality was checked with FastQC (https://www.bioinformatics.babraham.ac.uk/projects/fastqc/). *De novo* assembly of phloem sap transcriptome was carried out using SPAdes 3.12.0 [2] in “RNA mode”. The obtained *C. maxima* phloem sap transcriptome assembly included 96318 contigs. Sequences of *C. melo* pre-miRNAs annotated at miRBase [3] were downloaded in fasta format and used as queries for BLAST [4] searches for *C. maxima* assembled contigs containing related sequences. In order to obtain coverage values, primary reads were aligned with assembled contigs using Bowtie2 [5].

## CRediT authorship contribution statement

**Eugeny A. Tolstyko:** Methodology, Investigation. **Alexander A. Lezzhov:** Methodology, Investigation. **Anna D. Solovieva:** Investigation. **Andrey G. Solovyev:** Conceptualization, Funding acquisition, Writing - original draft.
