# Supplementary material for: Data on occurrence of miRNA precursors in the Cucurbita maxima phloem sap
Source: Data Brief. 2020 Jan 3;28:105083. doi: 10.1016/j.dib.2019.105083 (PMC7093818; doi:10.1016/j.dib.2019.105083)
Supplement: Multimedia component 1 [file mmc1.pdf]

Supplementary Fig. S1. Sequences of *C. maxima* phloem transcriptome contigs showing similarity to *C. melo* pre-miRNA (Table 1). pre-miRNA regions are shown in yellow, mature miRNA sequences are underlined.

```
>P3867
AATCCGTGCAATTCGAAGATAAAAGGCCATTCATTCAGAAACTCACTCAGTCATCGGAAAAAAAAAACAGAAC
AAATTCTTCTTCCTGTTCTTAGTTAATTAGAATGAGAGTTCCTGTTTCCAATCACCACTCATCCAAACCCTCC
TCTGCTCCTACACAAAATCCTAAGCTCGACTCCTCCCCCTCCCCCACCGCCTTCCGCCCCCCCACCACCACCG
CCACCACCGGTTACGAACCCACCTCCGTTCTAGATCTCCGCCGTAGCCCCAGCCCCGTGGCGCCAGATAACCC
TCTCTCCTCCGCCGACGACCGTCACAACAACAGCCATCCTCTGGACTGGGATGAACGCACTCTCCAGAACCTT
GACTGGGAATCCATCATGGGCGATTTGGGCGTAAACGATGACTCCAATTCTGCTCTCAAGAACACCACCGCCG
CCACCACCACCACCACCACCACCACCACCGACGACAATACCCACGATCATGTTCCTCACTTTTCCGAATTCCT
TCACTCCCAGCCGTTCGATCAAACCGCCCACCTCCTCCCCTCTGATTTCTTCCTCTCCGAACCATATTCCAAT
CACTCCGCCCCCATGCTCCACCAGACCTTCGATTTCGCCCCCAATTCCAACGACCCATCTCTCCATTTCCTCG
AAGATCTCGTCGACGCCGCCGATTGCTTCGATTCCAATGATTTCCACCTCGCTCACGTCATACTAGAGCGGCT
CAATCACCGCCTTCAATCCGCCTCCTCCGCCAAACCTCTTCACCGGGCCGCCTTCTTCTTCAAAGAAGCTCTT
CAATCTCTCCTCTCCACCTCCGCCACCCGCCATAACTCCTCCTCTTCCTGGCCCGACATCGTCCATACTATTA
AGGCCCATAAGGCCTTCTCTGCAATTTCTCCGATTCCCCTGTTTTCCCACTTCACCACCAATCAAATCCTTCT
CGAAGCTCTCAACGCTTCCTCTTTCATTCATATAATTGATTTCGACATCGGATTTGGTGGGCAATACGCCTCT
TTCATGAAGGAGATAGCTGATAAAGCAGAATCAAGAAACGTTGTTCCGCCAGTGATTCGAATTACCGCCGTCG
TGTTTGAGGAATTTGCGGTGGAGAGTCGGTTGACTAGAGAGAATCTCTGCCAGTTTGCCCAAGATTTGAAGCT
CCGATTTCATATTGATCTTGTGCCGCTTCAGACTTTCAAGACGTTATCTTTCAAATCGGTGAAATTCATGGAA
GGGGAGAAGGTGGCGATTCTGTTAACTCCGACCATCTTCCGCCGTCTCGGGAGTTCTAATGGCATAGCCTCTT
TTCTCGCCGACGTGCGATTAGTGTCTCCGTGCGTCGTCGTGTTTGTCGATTTCGAAGGGTGGTCGGACTCCGG
CAAGGCGTCGTTCAAGAGGAATTTGTTGGATAGTCTGGAGTTTTACGCCATGATGCTCGAGTCGCTGGACGCG
GCAGGGGCCGACGGCGAGTGGGTGCGGCGGATTGAGACGTTTGTTCTTCGGCCGAAGATCTTGGCGGCGGTCG
AAGGAGCGGGGAGAATGGCGGTGCCGCCGTGGAGGGAGGTGTTCCACTCGGTCGGGATGAGGCCGGTGGCGCT
GAGTCAGTTTGCTGATTTTCAAGCGGAATGCTTACTTGGCAAAGTACAGGTCCGAGGGTTCCAAGTTGGCAAA
CGGCATGCTGAGCTGGTGCTGTGCTGGCATGAGAGACCTCTGGTCGCCACGTCCGCGTGGAGGTGTTAGCTGT
TAAGAAGTTGAGCTCGTAAAATTGGATAACTACAAATCATGAGTAAAATTACAATTCAAATTCAAGAACATAA
CTCAAAATTTTTAAAATTTTGAAATTATATAAATTGGGTGGGGACTAAATTGTAATTTACCTTCTTTTTATTT
AGAATTTAATTTTCCCAATGTTATATTTACCGTTTTTCTTTTAACTGTTGTAAAAAACAATGTTATCTGAACA
CTTTTTGGATAAAAA
>P5314
GTTGTTCTTCTTCTTCTTCTTCTTCTTCTTCTTCTTCTCATCTTAGCTAGCTATGAGAGTTCCTGTTTCC
AATCACCAAATACCCTCTTCTTCTCCAAACCCTAAACTCAATTCCTCGCCCTCCGCCGTCGCCACCCCCACTC
CCACCCCCACCTCCACCCCCAATATTACCAACCCCTGTTACGAACCCACCTCCGTCCTCGACCTCCGCCGCAG
CCCCAGCCCTGTGGCGCCTGACCCCCACACCCTCTCCTCCGCTCCACTCCTCCCCACTGACGATCGTCCCAAC
GTCAACAACAACAACAACCCTTCTCTGGACTGGGATGAACAGGCCCTCCACAACCTCGACTGGGACTCCATCA
TGGGAGATTTGGGCTTGCACGATGACTCCAACTCTGCTCCCAAAAACCCAAACCCCGATCCCAATCCCAATCC
CAATCCCAATAATCATGTTCCTCAATTCCCTGATTTCCTCCACTCCCACCCCTTCGATCATCAAACCCATCTG
GTTTCCCCTGATTTCTTCCTCTCTGAACCCCCCTATTCCAACCACACCCCCACCTTCAATTCCTTCGATTTGG
CGGCTCCCAATTCCAACAACTTCTCCTGCGAATTCCTTGAAGACATAGTCCGCACCGCTGATTGTTTCGATTC
CAATGACTTCCAACTCGCCCACGTCATATTGGAGCGCCTCAATCAACGCCTTCAATCCTCCACCGGGAAGCCC
GTTCAGCGGGCCGCCTTCTTCTTCAAAGAGGCTCTTCAATCTCTCCTCTCCGCCTCCAATCGCCACAATCGCC
TCTCCTCCTGGCCCGAAATTGTTCATACAATCAGGGCTTACAAGGTCTTCTCTGCAATTTCTCCAATTCCCAT
GTTCTCTCACTTCACTACCAATCAGGCGCTTCTTGAAGCTCTTAACGGTTCGTCTTTCATTCATATTGTTGAT
TTTGATATTGGGTTTGGAGGGCAGTACGCGTCTTTCATGAAGGAGATTGCAGAGAAGGCAGAGTCCAGGAAGA
TGATTCCGCCGGTGCTACGGATAACGGCGGTGGTGCCGGAGGAATTTGCGATTGAGAGTAGGTTGATTAGGGA
GAATCTGTGTCAGTTCGCTCAGGATCTGAAGATTCGGTTTCATATAGATCTGGTGCCGCTTCGGACCTTCGAG
ACGTTGTCGTTCAAATCGGTCAAGTTCATGGAAGGGGAGAAGGCCGCGATTTTATTAACGCCCGCGATCTTCC
GCCGTCTTGGGAGTATAAACAGTATAGGGTCTTTTCTCTCCGACGTGCGACGGGTGTCGCCCTGCGTCGTCGT
GTTCGTTAACGGTGAAGGGTGGTCGGACTCCGGCGCGGCGTCGTTCAAGAGAAATTTGGTAGATAGCCTGGAG
TTCTACGCCATGATGCTAGAGTCGCTGGACGCAGCGGTGGCTGGCGGGGACTGGGTGCGGAGGATAGAGACGT
TTGTGTTCCGGCCGAAGATATTGGCTGCGGTGGAAGGAGCAGGGAGAATGGCAGCGCCGCCGTGGAGGGAGGT
ATTCCACGGCGCCGGGATGAGGCCAGTGGCGCTGAGCCAGTTTGCAGATTTTCAGGCGGAGTGCTTACTTGGA
AAAGTACAGGTTCAAGGGTTCCAAGTAGGAAAACGGCATGCTGAGCTGGTGCTGTGCTGGCACGAGAGGCCTC
TGGTTGCCACGTCGGCGTGGAGGTGTTGAGAGCTTAATTACCCGAGATATTGAGGGTATCAAAATTGATTAAA
AATTATAATTTACGATAATTGGGATTAGGGGT
```

>P7676

AAAAGAAGCCTCAATTCGATTGTCTGAAGTTCTCCATCTTCTCCATCAAAACTCCCTCTCTCTCTCTCTCTCT
AACAATTAGCCCTAAATTCCCCCAAAACAAAACCCCATTTCTTCTCTTAAACCCTTCTTCACAGAATCCTTCA
ATTCTCTGAAATACTCTCTACTATGGCTTCCCATTCCCCTAATCTCGAGTGTCGGATGTACGAAGCCAAGTAC
CCCGAGGTCGACATGGCCGTCATGATCCAGGTCAAGAACATTGCTGACATGGGCGCTTACGTCTCTCTTCTCG
AGTACAACAACATAGAGGGTATGATTCTCTTCTCCGAGCTCTCTCGCCGTCGTATTCGTAGTGTCAGCAGCTT
AATTAGGGTCGGTCGAATCGAGCCCGTCATGGTCCTCAGGGTCGATAAGGAGAAGGGGTATATTGATTTGAGT
AAGAGGAGGGTTTCTGAGGAGGATATTCAGGCATGTGAGGAGAGGTATAACAAGAGCAAGCTTGTTCACTCTA
TAATGCGCCATGTTGCTGAGACTGAAAATATTGATTTGGAGGACTTGTATATCCATGTTGGCTGGCCTCTATA
CCGGAAATATGGTCATGCTTTTGAGGCATTCAAGGTTATTGTAACTGATCCTGATACGGTGTTGAATTCTCTC
ACCCGTGAAGTCAAGGAAGCTGGTCCTGATGGACAAGAGGTAACTAAGGTAGTTCCCGCAATGTCTGAGGATG
TAAAAGATGCGCTAATTAAAAATATTAGAAGAAGAATGACTCCTCAACCCTTGAAGGTTCGGGCTGATATTGA
GATGAAGTGTTTCCAGTTTGACGGTGTTCTTCACATTAAGGAAGCAATGCGGAAAGCTGAGGCTGCTGGGAAT
GATGATTGTCCTGTTAAGATTAAATTGGTTGCTCCTCCACTCTATGTTCTTACGACCCAGACACTTGACAAGG
AGCAAGGAATCACTGTTCTTGAAAAAGCAATCACGGCCTGCACGGAGGCAATCGAGCATCACAAGGGAAAGCT
TGTAGTGAAGGAGGCACCTAGAGCGGTGAGTGAAAGAGACGACAAATTACTTGCTGAGCACATGGCGAAGCTA
CGACAAGACAATGAAGAGATTAGTGGCGATGAGGATAGTGAGGAAGAGGAAGACACGGGAATGGGAGAAGTCG
ATGTCGAAAATGCAGGTTCTGGGATCACCGAGTAAAGATAGTTGCAGGAATCATTTGCCCTTGTAAGTTGAAG
AAGGTCTGTAGTTCTGAGAGCTGTTCAATTCTTTCTTGACACAATTTTGAGAAAAATCCCTTTTTTTTTTGAG
GTAAGACACCTATAATACTTGAGTTATTACAATTGGGTAAATTGGAAAGTCTGTGTATTGCTCTTATTTAATG
GTTTTTTTCTTGATATTCAATGATTAAGCTTTGTGGAAGTCTTTGTTCAACATTCAAATGGTTGACTTTGACC
GTTTTAGACACACATTTGATTTTTCTAGTTATATATATAAAGTTAAGGGGTATTCTTATTAAAAAAAA

>P7902

CGTGTCCTCATCGTCTTCGATTCTGGTTGCTATAAAGGCCCCTGAACCGGCCATGGTTCTTCTCGCTCTGCAT
AGGGCTTAATCTCCGTAGTCTCTCTGTTTGCCTCTCATTTCCGTATCCTTAATCTTTCGCGGGCAATATCTCG
GCGTTGTTTCTTCTCAGTTATACACCGGCGGTCTCTAATTCGCTTGGTGCAGGTCGGGAACTGCTTCGCTCCG
GTTTGCGTTTGCCGACGGCCGGCATCTCCTAATCTTCTTTTTCTGTTTTTCTCTGCTTCTATTTTTTTACTTT
TTGCTTCATTTCTTTGCCGCCGTGTGGTTTAGCTTGTTCGGGAAATGTTTGGGCGAATTGGATCCCGCCTTGC
ATCAACTGAATCGGAAACCGTGGTGAAGATTGCTTGGATTCTTACCTTCCTACAGATCTCAGGTACGGAGTTT
CTCTTACTTTCGAGAGAGAACGAGTTAATTTAATTGTTCGCATTTTTTCATGATTGGAGTTGTAGAACTTTGT
TGCTAGTTTCGATTTCTCTCCATGACTGAAATTAATAACCGGTTTGAAAATTACATAATAGATCTGGTCTGTG
CTCTGAATCTTATTTAATTATCTTTCTTCCTTTGTCGTCCGCTTTCGGAGTTAACTACTGAAGAGCTTTAGCT
GTGTTTCGCATTGAGGCTGATGTTTGTATATCATAGAGCTAGAGTTATTGTCGTTGAAGTTATTTGGTTTCTG
TTTATTGCTTCCATCCATGAAACACAATGACAGATGTTACATCTAATTTTGGACATAAAACTCCGATTCTAAT
CCGTATGGAATAACGGAACTGTTAGAACTTTGTGTATCATAGATCCAGATTTATTATCGTTGAAGGTATTTGG
TTTCAGTTTATTGCTTCCATCCATGGAACACAATGAGAGATGCTACTTCTAATTTTGACATAAAACTTCGATT
CGAATCCGTATAGAATAACGGAACTGTTAGAACATTATACTTCATATTCTACTCCTGCTAAAAAATCAAAGGT
CAAAGAAAAAAAAAAGAAAAGGAAAAGAGAGAGATGACAATAATCTTTGAACTGCTGTTTACCTTGTAATAGT
GTTTGGTCCAATTAATTGATCTGTTTGCTGAGTACATTTTCCGCGAATAATGAGTTTGACAAAAAGAAAGAAA
GAAGTGGCGACCTGTTTGTAAGTTGTAATCTTCTGGTAATTCTTGTATGACTGTAGAATTCCCCGCCAAACCA
GGTTTCCTGAATTTTTGTTTCCTTCGTTTGCATAGTGCAGAGTAGAACTAGAACAATGAAACTTGTATTCCGA
AAAAGGGGGGATTTGTTTTCATTTGTCGGCTCCTGGGCCGTCTGGCTCCATCATTCCATGATAATAGCCTGTG
GAAATGGAGACATTCCGATTCCTTTCGTTCGGAAATTAAGTATAAGTGAACTGATTGTGTTCCATTGTAATAG
GAAATAGCCAGTAGGTTTTGTGGTTGGTAGGTGGTTCACCATAAAAGC

>P8823

AGAATCTCTATCTCCTTCGAGCCAAAATCAAGCTTCTCTCTCTCTCTAACCATAGCCCTAAATTTGGCCAAAA
AGAAAACCCCATTTCGTCCCCTTAAACCCTTTTTTGTCACAGGAATCTTTCTAACTCTCTCAGACCTTCCCAT
CTCTCAACCATGGCTTCCCATTCCCCTAACCTTGAGTGTCGGATGTACGAAGCCAAGTACCCGGAGGTAGACA
TGGCCGTCATGATCCAGGTCAAGAACATCGCCGACATGGGCGCCTACGTCTCCCTCCTTGAGTACAACAACAT
CGAGGGTATGATTCTCTTTTCCGAGCTCTCTCGCCGTCGTATTCGTAGTGTTAGCAGTTTAATTAAGGTTGGA
CGAATCGAGCCTGTCATGGTCCTCAGGGTCGACAAGGAGAAGGGCTATATTGATTTGAGTAAGAGAAGGGTGT
CTGAGGAGGATATTCAGGCCTGTGAGGAGAGGTATAACAAGAGCAAGCTTGTTCACTCTATAATGCGCCATGT
TGCTGAGACTATGAACATTGATTTGGAGGACTTGTATGTCCATGTTGGCTGGCCTTTGTACCGGAAATATGGT
CACGGTTTTGAGGCATTCAAGATCATTGTAACTGACCCTGACTCGGTGTTGAATTCTCTTACCCGTGAAGTCA
AGGAAGCTGGTCCCGACGGACAGGAGGTAACTAAGGTAGTTCCCGCAATGTCTGAGGATGTCAAAGATGCACT
AATAAAGAATATTAGAAGAAGAATGACTCCCCAACCCTTGAAGGTTCGGGCTGATATCGAGATGAAGTGCTTC
CAGTTTGATGGTGTTCTTCACATTAAGGAAGCAATGCGGAAAGCTGAAGCTGTTGGAAACGATGATTGTCCTG
TTAAAATTAAATTGGTTGCTCCTCCACTCTACGTCCTTACTACCCAGACACTTGACAAGGAGCAAGGAATCAC
TGTTCTTGAGAAAGCGATCATTGCTTGCACCGAGGCGATCGAGCATCACAAGGGAAAGCTTGTGGTGAAGGAG
GCACCTCGAGCGGTGAGTGAAAGAGATGACAAATTGCTTGCTGAGCACATGGCGAAGCTACGACAAGACAACG
AAGAAATCAGCGGAGACGAGGATAGTGAGGAAGAGGAAGACACAGGGATGGGAGAAGTTGACGTCGAGAATGC

AGGTTCCGGGATCACTGAGTAAAGATTGTTGCAGGAATATAGATAAGAAGAAGGTTTTCATAGTAAATCTTTC
CCCCTTGAGAGTTGAAGATCTGAAGGGGTCTGTAGTTGCGAGAGCTATTCTTGACACAATTTTGGGAAACCCC
TTTTTTGAGGTAAGACGCCTATTCTTGAGTTGTTACAATTGGGAAAATTGGGAAACCATTAGTTTCAGATATT
ATTTGATAATACCATTTTCAGTATAGTTTTTTTTAAAAAAA
>P10713
TAAAGGAAATCTACAAAGAATTTTAGTCAAATTCAACAAGTCTGTAAAACTCATTTTGAAGTCCAATTTTAGT
TTAGGTTTATAAAATTATGGAGAAAAAGTGAATTACAAAACAAACAAACAAACAAACAAAGAGTTACAACACT
TCCTTGCATAAAGTGGTAGTTTAAAGGGGAAAACGTACTTGAGATGCAGAAGCCTTGCCGGAAAGTTTGAGAT
GGGGAGCGGCGGAGGGCGGGGGGTTTAATAAATAGAAGGGATTAGTAGCTAAAGGAGGCATCCGAAGGGATAG
CATGATCCAAAATTACGTAGAAGAAATTGAAGGATATAGATAAGTTGGGATCAATGCGATCCCTTTGGATGCT
TCCTTTAGTTCCACACAATAGCCTTTCATCTTCTTTCATCTCCATCCTCTAACTCGGTTCCTAGGGGAACACT
TCAATATAAAAAGACCCACTTTAGTGCTTAACCAACCTTCCCTCCCCCTTTTATTCATTTTTTTTTATTATTA
TTTATTCTCTCCCTTTTAATTATAACTAAACTTATTCTAATCCTTTAATAAGTATAAAAAAATAAATATATCA
ATACTTAACTTTATTTTATTAATTTTAACTACCTTTAAAGAGTTTTATTAGCGTGCACGTTAGTGAAAATTAA
GTGAGAGTGTAAAAATTACTACCACTTATTTAAGATATTGACATTAATTTATATATATGGTCAGAAATTATAT
AATAATCTTGGAAAAGAAAGAAAAGAGAATAGATTTTGAGGTTTCCCCCTTTTTGTGTACAAGGTGTTGGTGG
AATTTAATGGGTTTGAAAAACATTCCAATTTTGGGAAAATTAAGTTTCGTTATTCCCAATGCACTCAACCCGT
TTGTTATTTTTTCCAGAGAAGGAAGAACAGGAAGAGAAAGAGGAATGGCATGAATAGGCAATGGAATTGTGTT
TATAATCCATGTGATTATGTTTGATTACGTTGAGCCAAAAAAAACCCAAAACCCATTTCATTGTAGTATCTTA
TGGAACACACATAGTTGTAAGTCTCATTATTGTTAACAAATGAATGTTTTGTTTGTGTGATGCAGGGCATTCA
GGCTTAGTTGTGCAGATGGGCATTAATAGTGGAATTTATTTTGAAAACTTTTTGAGTAGAGAAAAGAAAGTGA
CAAAAATGGTGGTGTGATGGGTTTTTGAAAACTTCCTCCATAATGCACAATGGTGGGTACCTTATAAATGGTG
CACATGGAAAGACGATGATGATGATGATGTATGGTGCTAGATTTTGTCCAC
>P12201
GGGAGAGAGGCGGATGTTGTCTGTTTGCGTTCAATTGAATAATTATGTTTGTTCTTCATCTGGTTATGATACT
TTCGTGACCTCTACAGATCAAAGATAAGTTCAGCTGCTGTGGTTGGTTGTATGATTCCACTTATGTTGGGAGT
ATGCTTTCTTTGTCCTTCAATGGACTGCCTTTGGCACAAGTATCACTATTGCCCCAGCTGCAAGGAAAAGGTT
GGCGATTTTGAGAAATTGGATAAGTGTGCTGTGATGGATCCTTCAAACTGGACACAGCAAAGCCTTGCGTTGC
CTGCTTGATTTTCACGAGCTGCTACTTTTCCACGCGGTTATTTTCCGTTGTGTGCAGTCAGTTTTTGCTCTAC
TCTTGTGCTCTTGTTGAGATATTCTTGTTACACTTAGGAGGGATAGGCAAAGATTGGAGATGTGTGCATACGT
ACACTCGTGATAGAACCAAGTACATATTTTTATTCATTCACCTTACAAGTCATTTCATACACCTTTTGTTCAT
TCACCTTACAAGTCTAGATTGCATACGTTTGTCCAATTAATTTCCCTTGGCTGCACACTATCTGCATCCCCCT
ATCGTCATTTGTTTTTAACTTCTCAAATTACATTTTGACCATGCATCTCCCCCTTCGGACGAATCGTTCATAG
GGGCTTCTTTTCCTTCTTAATTTGGTTTTCCTGCTTGGTTTTGAGGGGACTTGAACTCTGATCCTTGATGGTA
CTATCTGGTATCACACAAGTAAGCCGTGAAGAAACATCTTGGAAAAGATGATATACCTGAGTTCATTGGGGTG
CTGCCCAGATGCAGATGAATCTAATTGGAATGCTGCGGTTTTGAATCTGGAAGCGAAGGCCCGGCATGAGAAA
TAAGAGCCGGTCTCGATAGCCAAAGAGACTGCCTGCAGGGGGTATGCCACGGCAGAACGTCCGCTTAAAGACG
ATGGCAGCTTTACAGATCCCTTTCATGGAGTTGCAGGCTGCAGGCTGCAGGCAAGTCATCCTTGGCTACAATA
CATGACTCTCCATTCCCATGTTGAACATTACTTCCTTTCTTCCCGAGTAGAAAAGGAATTAAAGCCCCCTTGT
GTGCATTCCTTTTGAACCAAAAGGAGAATTTGTGTATGTGTGGATTGAAGTTTGGTTTTATATGTTAAAATTA
GCGGCAGCTCCACAACAACATGCTATGCACTCT
>P17547
CTTATTTGCATAAATGGGTCATTTTTTTCTTATGAGTTTCTTGACCAAGTTCAGTTTTTTCCTCGTACTAAGC
TAAATCCATTTGATCTTAACTGGGTTTTTTGTTTTCTTTAATGGGTTCGTCTTTGATTTTTGTTACATGGAGC
TTATTTGCATAAATGGGTCGTTATTTTCTTATAAAGTTCTTGATCAAGTTCGGTTCTTTCTCATATGAAGCCA
AATCCACTTGATCTTAATTGGGTTTTTGGATTTCTCATCTGGGTTGTTCTTAGATTTTTGTAATATGGAGAGT
CTGGTGTAAAGATCTGATCTTTTTATGGTAGTAATTAGGGTTAAAGGCAGAGCTCCTTGAAGTCCAATAGAGG
GTTATGGCAGGGTGATTGAGCTGCTGAACTATGGATCCCACAGCCCCTATTTGATTGAGCTGCTCATAGGCCT
GTGGCTTGCATATCTCAGGAGCTTCATCGCCTGTTTGGTTACACCCTTGTTTGGATTGAAGGGAGCTCTACAT
ATTCTCTCTAATAATCAATATTAGCTTTTGTTTGTTCATATTCATTCTCAGCTAACCCTTTTATGCCTTGATT
TTCTCCATTGATTTTGATGGATCTTTGCTTAACTTATGTTTTGGATTGAAGAGTTGATCTTTGCAGGCAGTGA
AGGGCTGTTTTTCATGATCTATTTCGATTAGAACGGCGTGGGATCTGTGCGACGTTCATATGTTCTTTAGAGT
TGGGAGTACGATGTTGTTGTTGCTTGGTCGCTGTTTGATGTTTCTGGACATCCTTTTTAGGGCTGCAACTTCG
AATTATTTCTTTCAATTTTTTTTGTTTTTTGTTAATAGTTTGGGATACAAATTCCTATACATTCCTATGATGA
TAATACATAACAACATTATATTTGTAGGCAAAAAAAAAAAAAAAAAAAA
>P18642
CTTCTCTCTGTACTTTTATCTTTTTTCTGTAAGCCATAATATTAATGTTGTCTAAAAGGTTTACTGGTTTAAT
CCTTGAAGCTTCCCTTGAAGGATATAAGTTGTCCGTAATTGTTTTTAACCTTCAAAAGATAATTAAGAAACAG
AAGGCCAGAAATGAAACTACTCCGCCAACATGACTTCTAATATAGAAAAGGAAGTTGGTAAAGATTTTGTTGA
AGGGTTCAATTTGAATCGGATTATAATTTTTAGTTTTCAAATTCATTTGACATTGAATGCAAACTTTCAAGAA
CTGGAAAGAAAACAATGTGTTACTGAAACATAAACAATGATATAGTTAGTCTTCAAGCAGCATGTAGGAAAAT

GAAAGAAGAAGCTGAAAGAGAGAGAGAGAGACTAAAATTGGTATTAAGATACTCAGTTAAAGACTGGTGACGG
AGAGAGAGAGAGCACACAGAATTTCTAGCATGACCATGCAAGAAAAGAACTATGTGTGCTCCCTCTCTTCTGT
CACCTTCCTTGGCACTTCAGATAAAGATTACCTTCTGTTTGTTGAGCAATTTTTCCTCCAACTTTAATTCCAC
CGGAAAAGGAGGATAAGTGGCCATAGAAACACAAATTTGGATGGTTGATCTGATTTCGTACTAATTACTGATA
TTTATAAGAAGGTTACTTGTGCTCTGATTAAAGAAAACATAAACCTCTGCTTTAAACTCTTATTGGTCATTCT
ATCCTAAAGTTCTAATTGGAAGCATTTTTACCACTAGGTTGGGGAATGGGAATTGTTAGACGTCAAGGAACTG
GATTATTCTTAGCCAATTAGAGCCGATGTGATATTTTCGTTTCTTATGCATTAATCACATGTGTAATAAAATT
GTTG
>P19669
TTAGATGAGGGTAGAAAGTGGAGTCCATGTCACAATGCCCATACCCAACAAAAGCCTTTCCTTATTACGTATA
AAAGGACCCCATGAAAGTTAAAAATAATGCATGGCCAAATCAAATCTCAATCAAACCCCTTCTCTCTTTCTTT
CTTTCTTTCTTTTCGTTCCATCTTTACACTTCTTTTCCTCTCTCTCTCTCTCTCTTTATGAACAGTTGAGGGG
AATGTTGTCTGGTGCGATATCTTTCATCCTTTGAATTTGAAACAATCAAACTCTTTTAAGTTTTGAATGATTT
CGGACCAGGCTTCATTCCCCTCAACACACCCCATTAAATCTTTCACTTTTTTCTTCAGTTGGGTGGCCTCGGC
AAGCCTCTGCTGCCCGTCATACAGGTATGCTGGTTATATACTTATGCAGGAGGTCTGTGAAGGCTGTCCTAAT
TCACCCAAGTTTTCATGGCTGTTTTACTGTGTGTTTTTTGATTGTGTCGGGAAGTTTATTTGAAAGAAATAAG
GTGGAATCTACTGCTGTTTCTTTGTTCTTCTGTTGTGCTTATATCCAAGATGAAGTTTTGTTCTTGTTGAGAA
ACTTTCCCCTCCAGCCCAAACTATAATTGTAGCTACACAATAGAATAAGAGGGGAGGGGAAGGGATTCCAACT
CCAGTCAATTTTGTTCGTTAGAAATGATTTGGAAGATGTAAACCCGAAGGGAGTAGCTGTCAAGAAGCGTACT
GTAGTGTGCTTGATTTTGTTTCTAACGGGTGCCACTTTGGTTCATTCATCTTACAATTGATATAGCTGATATG
ACGGAAGAATATCAAATCATTACTAAACTAGAGAAACC
>P20012
CCCTTTTCCTCATCTTCCTGGGATTCATGATCAGTTTCTTCATCTTCGTATCATTATATTCTTTAAAGGGTCT
CTTTTTCTCTCTGATTTTAGGGTTTTTTCGGTTGTTGGCGTGATCGGATTTGATGTTTTGTGAGGGTTAAGAG
AGCTTTCTTCAGTCCACTCATGGGTGGCGGTAGGGTTTAATTAGCTGCCGACTCATTCGTTCAAATACTGAGT
CAAAAACCCGACTCTCCGATTCAGTAAACGAATGAATGATGCGGGAGACAAATTGAATCTTAAGCTTCCTGTA
CTTGGACTGAAGGGAGCTCCCTTTTCTTTTACCCTTTTGATTTTATCCGTTTTTTTTTTTTTATCGAAATCTTC
GTTTTATCCGTTAAACGATCGAAGACCCAATTTCATAATTACGGTATTATGTTGAAAAGATGTTAAAAATCTC
GTCTTTTTTATGGTTTTCACCAGCATTGATGGTCTGCTTGGAGTCAGATTTGAGTAGGAATTACCCATTTGAA
GAAAGTTTTGAGGTAAGATAAGTTAAGGTTGATTCATTATTGGGTTAATGGGTGGATTAGGGTTCTTGAAAAT
TGAAGGAATATTTTTTACAGAGCAGTAAAAAAAAAGGGTTTTTGCTGGACAGACACGAGCTTCGATGCCATAG
CTTCTTGGGTATTGGCCATATTGCTGCAACAAGTTCCCAGACATTTTATGTTACCACCACCCTCTTCCCATTC
TTGGGTATCGGTCTTAATGTTTTGTTTTTGTTTTTGTTTTTGTTCTTCTCTTTGTCTGTGGTTAGTTTCTTTT
TTGTGTTGTTTAAGGTGGATTTAAG
>P20793
TCTCTCTCTCTCTCTTCCTCAGATGACTTGGGTTAATTTAAGCCGTTTCTTGTGGTTTTAATTGATGGATTTG
TTGGTGGGTTTCAATATTTTGAATTAGATTGTACAAAGGAATGAAGTTAATTTATTAGCTTATATGGAGTAAA
TTAAGATGTGGGTTTCATAAGTATTGTGTATTGTGTTTATGTGCAGAAAAAGAAGGAGCTCTCTTCAGTTCAT
CCCGAGACAGTAGAGGCTCAAAAGGTTGCTGCTCATTCGTTAGTTCAATAGCTCATTATACTCAAAATTTCCA
CCGTTGAGATGCCGAAGCTGTGAGATGAGCTCCTGATCTAACGATGGAGGAGCGGTCTTTGATCATCACTGTC
ATGTATTGGACTGAAGGGAGCTCCTCCTTAATTTACTTTTCACACGCATACTACAACTAACTCATCCTTTGTC
GAGAATGATTCAGTAAGTAATAGTTAAAAAGCTTGTTTTTTTTAACTTTTTACAATGACCCTTAATTTGTGTA
GGAATATCTGTCTGTTAAATTTGTGTGGTTTGATAGAGATTTGAAGAGAGCAGAAGCAGTTAAGGAGGGTAGC
TTTCTCAGGGTCCAAAGCTTTGGGCGATGTGTTGCAACTTTGTGAATCTTCCTTTTAACACTAATTCTCTTTG
TGGTCTTACTATCATTATTTTTTTTTTCTTTTTAATTCAAATATTGTTGAAATTAAATAATAATTTAAATTTT
ATTTGTTTAGCTATAAACATTAATATACTTTATGCATTAAAATTTTAAATTTAGATGTGAAAATGAA
>P21134
CCCTTTCCTTTCATCTTCAACCCTTTTCCTCTTATGAATTTTTCCTTTCATCTTCAACCCATATCTAATGGCT
CGTCATCTACCTCTAGGGTTTCTAACCCACATGGGCTTTGTTCTTCCTGATCTAAACTGGGTTTTTGCTCTTC
TTTTCTGGGTTCTTCTTGGATTTTTGATTGATTGAGTGTTTGGTTTAAAAATTTAATCTTTTTGGGATTAGGG
TTACAGGCAGAGCTCCTTGAAGTCCAATAGAGGGTTGTGCTAGGTTGATCGAGCTGCTGAGCTATGAATCCCT
CAGCCCCTATCCCTATCTCATCAATCATATCTAAAGCTGATAGGCCTGTGGCTTGCATATCTCAGGAGCTTCA
TCAACTGCTTTTGTTAAATCCTTGTTTGGATTGAAGGGAGCTCTACATATTCTCTCTAATCATCTTGTTATTA
CCTTTTCTTATGCCCTAATCTTCATTGACTTGAGTTTCTTGTTCCTATTATTTGTTATGATTTGATGAAGGTC
TGTGATTTTTGCAGGGGATGGAATGGTTTCATTGTTGGATCTTGCTTAGATTGATGGGATCTTTTAGAACATG
TTGTGAAGAGATGAATGTCTTTTTGTAGTGTTGGAGTGTACGATGTTGCTTTGTAATATAGCCATTTTCAGGT
TCTTGGAGATTTGCTAATGGAACCCTTTGTTGTTATTAAGCAATCATTCAGATCCTTTAGCTTTTGGCTATTG
AGTATTGATGAATAGTAATGGCAACGATGAACTATAATCTATCCTATTTTCTCG
>P25386
CATTCATTCACCCTCTCCATTCCTTTCTGATATATGCTTTTTCATATATCAAATCTGCCTGGCTCCCTGTATG
CCATTTGTAGAGCGCTCACCGTCTTACCCGATGACCTCCGTAGATGGCGTATGAGGAGCCAAGCATATTCCGT

GTAGATCGGGATTCAAATCTTTTATCTTTAACATCACAACTTACCCGTCTTTTGTTTTTAATCCATCTATGTC
TTAATCAGCTCAGCTATGCTCTAAATTGGTAATTCTCGACCTAACCCTTCTCAATGCTCGTGCCCTTTTCTTA
TTGCAGCTACCCGAAAAACCCTAATTTCAAACATTGGCATTGAAGAGCAAGCGACCGAAGATCTGATGGGATG
AAATCTGATCGTGTGGTGATGGGTTGTTCAATATGAAGAGAGTAAGGTAGATCTGGCCACAGAGGATCCAAAT
ATATACATATATATTAGAATGGGAAAGAATCCGAGTGGAAAGAGCTTGAGGATTCTCTCATAGCTAGGGTTAT
TTGGATAGCTATCTTAGCCTAATGGCTAGCTATATTCGACTACATGTAGTGTAATAATCAACCATTTATCTCA
ATAAATCTTTTTTTTTTTTTTTGTTCGTTTTCCATTTTCGAGCTCTTGCTCTTGCT
>P25784
TTCTGAAATGGGCGTTCTCTGGACTGAGTGGGGAGCAAACGCAATATCCTTAGTGTTGTTGGTGATTTAGTGA
TTGAAGCTGCAAGATGACCTGATGGAAGAAACCAACCTTTAAGGCGGCCACCGAATCATCATCGCTAACAAAA
TATTTTACCCTTCCCCAGATCATGCTGGCAGCTTCAACCTCTCAACCCAACACATGTTGAAAGACCAGCACAG
ACAGAGAGAGCTAAACGAATTATTACAGATCCAAAGTTAACAAAGCTTAGATTTGAAGACAAAGGAAAGACAA
ACAGGGCTATATGAGCATGAACTCTCGAGTCATATTGAGTCAACTATCCCCTTAACTGGCATTTGGCAGAGGT
TAGAGAGAAGTTTATATAATGCGGCTTTCTGTTGGGGTTTACAGCTATTTTCAGCTGACCCATGAGCGTTCTG
TCCAATCCCATTATCATCATAATGCTTAATTGCTGATATAAAAATACAATATTAAGTAGATGTTTGGCCATAG
CAGCAATTATGTGGTGGTGATCCATGTGAATGCATAACCAAGTAATGCAGAAGAACAATAGATATCGATGAAA
TGTTCCACTTAAAAAAAAAGGCCAGCTGGGTAGGGATCAGGTT
>P29894
CATTCATTCACCCTCTCCATTCCTTTCTGATATATGCTTTTTCATATATCAAATCTGCCTGGCTCCCTGTATG
CCATTTGTAGAGCGCTCACCGTCTTACCCGATGACCTCCGTAGATGGCGTATGAGGAGCCAAGCATATTCCGT
GTAGATCGGGATTCAAATCTTTTATCTTTAACATCACAACTTACCCGTCTTTTGTTTTTAATCCATCTATGTC
TTAATCAGCTCAGCTATGCTCTAAATTGGTAATTCTCGACCTAACCCTTCTCAATGCTCGTGCCCTTTTCTTA
TTGCAGCTACCCGAAAAACCCTAATTTCAAACATTGGCATTGAAGAGAAGGAATATCACAGATTCACAGACAC
AATACGTCTTTTATTTTAGGGTTTTCATCATTTCCCTTAAATTTAGGGTTCTTGTTTGGAAATTGAAGTTATT
TCACTGTTTGGCAACGGTTTAAAATGTATCCATGTTCGGGATTTGTTCTTAGCATTAACCAGATCTCATGTAT
ACCA
>P29987
CTCACTCCTCTCCTCCAGTCTCCGCCTTCTCTCTAGGGTTCGATACCCTAATTTTTCTTTTCTTCGTTCTTCA
GAAATGGCTCTGTTTGTATTCTTCCACAGCTTTCTTGAACTGCATCTTCCTGAGCCAAATTCGCTCCTCTCTC
TCTCTCTCTCTCTCAAAGCGTTCTTTGTTCATTAACTTTTGCGGTTCAATAAAGCTGTGGGAAGATACAGACA
AGGTCAACCAAATCGGTAATCCACAGCGAAATTCGCCGCCTACAATGACCTTCAATCGCCGGCGCCGTCGGTA
TATTTCTGTAACTTACAAAATCGTATGATGTAATTTTCCCCTTTCTTAAGCTGTTTTTGTGCGTGTTTGTTTG
TTTGTTTCTTAATTTGCCTTTTCAGAAACGGTAACTTTTAGGTCAATTTCTCCCTGAATTTACTCGGAAATGT
GAGGTTTCAATTCGATTTCGATTTAATACCTAATCTTCTCCGTTGATAGCAGAAACTATCTTGAATGGATTCA
AG
>P31502
CTTATTCTCTCTCTCTCTCTCTCTCTCTCTAAGCCTCTGATTTTGAGCAGATGGGAGAAGAGAACATGAAGAG
TTCTCTTTTGATTATTATTGCAATGAGATAGGCAAAGGGGTGTTTGGAATGGAGTTTGTTCCGAAATCTTTGT
GTTTCTTCGTTTAAAGGGTCGTTCTTTTTCGTTTATTTTGTGTCATTTTTTTATCCTTTGCAAGGTTTCTTTC
TTATGAGGTTGAAGATTGTGTTATTCTATGTTTTTTCGAACTAAAAGTTGTGTTCTTCTTCTTCTTCCTTCTC
GTCTTTGAAAAGTTCGTCGTTTTGTGTTGATTGAATGGAAAAAATGATCTCGGACCAGGCTTCATTCCTCACA
CGTAGCTTTTCCTTGGAAAATTTTGGATATGGGAAAGATGAAAGACCTTTGATCAACTTCTGGTAAGCTCTCT
TGAATCTCTCACGAGGATCATATATATATATATATATATATAG
>P37982
CGCCTCGCCCGCCGACATGGTGCTCCGGCAACCGGCAGTAGCACGGGAGAATCCGTCAAGCTCAGGAGGGATA
GCGCCGTCGAATGAAACACCACCGGAATGTTCATTTCCGCCGCCGCTTGCCGGCGCTATCTATCCTGAGTTTC
ATGGCTTCTTCTTGCTTTTTCCAGCTCGGTTTTTTACAGGCATTAGAGGACACGTGGACGGTCGGATGGAGAG
GAGTGGAATGAAGAAGGTTAAATAGCAATGGGTACATGCAAGACAACATTGCGTTTTCTTTATAAAATTAATT
CCAGAAATTTTAGATAATGTTGAAATATGGACTAAATGAAAATTAATTATAAATAATAGATTATATCCGTCG
>P38121
AAGCCAGGAAAGCAAAAGAAATGAGAGAGAGAGGGGAGGGTTTAGTTTGGGAGAATCTGTAAAGCTCAGGAGG
GATAGCGCCATGGTTCGTATCAATAATAATATTATTACACTTCCATTTTCTTTCAATGGCGCTATCCATCCTG
AGTTTCATAGCTTCTTCTTACTCACTCGTCTTCTCCTTCCTCTTCCTCTTCCTTTCATAGCCTCTCGTTAACG
TTTTAATAAAAATCGTTTTTTAAGCATTTGAAAAGTTATTCTAAATGGAATGATTATGTCATTACATTCTTCT
TCTTACGTTCATAATCGTTAAATTTTCTATTGATTGTTTGTAACGATTGGGATGTTGATTATATCACCCG
>P44166
ATTTTTCTCTCTAAAAAAGTTGGAGTTTTTAGGGTTTCGGAGACGAAGAGCTCGTTTTGTTTTGAGGGGAATG
TTGTCTGGCTCGAGGACACTTTTCGTTAGCGTTTGATTTTTGTGTATATATTTAGTGTCGTCGGACCAGGCTT
CATTCCCCCCAATTAATTCTACGGCCTCCAACACCGACACCGACACGGGTTTGTGTTTTTGATTTTTAATTCT
ATTTTTGGTAAAGGGTTTATGAGAATATGATGTTGTGTTGTGTTTGGAAGTGAATTAATGAAGATGGAGTGGC
G

>P45184
CTTTGGCTCTCTTTTCTCTCTTTTTGCCCTAATTTTTGTTTGGTTCACTGTTGTCGTGCGCTTATATCAGTTC
AAGCTGCCAGCATGATCTAACCTTAATCTCTGCTAATTATGTTCGAGGTTCAAGATCAGATCATATGGCAGCT
TCACCTGTTGTTGGCTGCACGAAGAACTTAGCCCTGAACACAAAACCCTAATCACCTTTTTTCCTTAACAATT
TCTTCATTCTCACCCGTTTTCCATGCATAATTCCTAAAAAAGGTACGAAGTTTTTGTTCATTCTCT
>P48351
TCGTATCATTCTGATTTGAGGAACGCTCGTGTTTACTACATGCCTGGCTCCCTGTATGCCATTTGTAGAGCTC
ATCCGACTCTCCGATAGGCCTCCGCAGATGGCGTATGAGGAGCCATGCATATACCCAATTTCACCTTCCTCAC
TCCATTCTTCCATTAGGGTTTCATCAAACCCACCAAAAAAAAAATTAGGGTTTTGGTCAAATTTTGAGGGTTA
AAAGTAAAAAAGTGTTTCTTTTGAGGGATTACTTATTAATAA
>P48844
CTCGATCCAAAAAACAGCCAAAAAAATCATGAAAAACATTAATATCTATAAAGCATATCAAAACCTCCATATA
TTTGGTAGAACAATCAGAAGTATATCAATCACCGCCAAGATCTTCATCATATTGGGCATGACATGAGAGAAAG
AAAGGCACACTTAGCCAAGAATGGAATTGCCGGCCACAAAGAGGAGGAAGTTGCTATCAACATTATCATAAAA
TAATATCAAAATCCATTGCCGGCCGGCAATTCATCCTTG
>P49298
ATGATGCATGAACAGATAGTGAGTGGAACCTGAGCCAAGAATGACTTGTCGGCCGAATTTTGATACCCATTTT
TTCCGTCGGCACCGTCTTCTTCGGCTCTCGCTCCCTCTCGATCTTTCTCATGCTTCATTCTCGATGATCTTCA
ACAGTTTTGTAGCCCAATCCTAATGATTTTGCTGCTATAGATTCATAAGAAGGGATGGTTTCTTGAGATCGAA
GAATAATGCATGAAGAGATGGTGAGTGGAATTTGAG
>P50997
GTCAGTGGAGGCAGCGGTTCATCGACCCCTTCTTGGGGTTTGTACTGATTTGTATCACAAAAACACGAACCGG
TCGATAAACCTCTGCATCCAGCGCTCCCACCCTTGTTGGTTGTTCGTTTATAATTTTAGGACAACTGGGAACA
GTGAACCTCTTTTGCAGTTCGTTTCTTTGACCATCTGGGACCCCTTTGTGTTTCTGTCTGCATCTTGATTAGG
GGGGCAAAGGCACAACAAAATTTTCT
>P53142
CTTCCTCAGTCAAAACTCTCTACTCTGCTATTTTTTCTTCTTTTCTGCAGAGTGAGCTTTTATTTTATAATTT
TTATTGGGTAAGGATCAGGTAATCTGCATCCTGAGGTTTAGATCAGAGTAATCAAATTTAAAAAAGAAAAAAA
GAAAAAAAAAGTAAATTAGGAGTAAATGGCTCGTTGAAAATGGCTCTTCTCCTAAGCCCTACGACTAAATTCG
GAGTTGAAAGGTTT
>P53263
GAAGCATTGACAAGTGACCAATAATCAATTGCCATTATATACATACCCAAACCCCTCTCCTTCTCTTCAACCT
TCCTCCTGTCTTCTTCTTCCCTTTCTTTACCCTTTCGGTTTCTTTATGTGTTTGTGCTTGTTCATCAATGACA
GTTGTTGGTTGCTGGTGTAGCATTATCAAGATTCACATTCAAATGGGTTCCGGATTTTCCAGGTTTCTTTGCA
ATTGAGAATCTTG
>P53520
TCCTCCACTCTTCTTCACCATCCTCATGCTGCCTCTTCCAGGTTTTTGAGACAGCCCATGTCCGAAAAGGTGT
GTAATGAAGGATATATTGCATGAAGAAATGGAGAGAGAGAGCTTCCCTCAGCCCACTCATGGAAGGACTCAAA
GGGGTTGCTGAACTAACTGCCGACTCATTCACTCAAACACTCAGTACTATTCTTTCCTCTCTACTGTGACTGC
GTGAATGAAGCG
>P55325
CTCCATTTCTCGCAATCTCCGAATCTCTTTGATTATATATTCTTCTCCCACGTTTGGACACAACGCACCAGAA
ATTTATGGCCAAATTGTTAGCCTTCTCCTGCATTTGCACCTACACCTTCAGCCTTGCTTCTTCTTTTCTCTTC
AAATGCACAAGGCCACTGAGATGCAGGTGCTTCTGCTAGTGAATGCCATCTTGGCACTCTCTCTCCGCCACCT
CCGC
>P60916
GGTTCATCGACCTCTTCTTGGGATTTGTAATGAATCGAACGCAAAAACACGAACCGGTCGATAAACCTCTGCA
TCCAGCGCTCTCTCTCGCTCTCTGTCATTTTTCCCTCCTTTTGTTTGGAGAAAAATTTAGGACAACCGGGAAA
GTAAAACCTCTTTTGCTTCGTTTCTTTGACCATCTGGGACCCCTTTCTGTTTCT
>P69980
ATAAAGTGTATACTAATGCTTTTGCGTGCTCACTTCTCTCTCTGTCAGATTCGATAGATGGATCGGGCTCCGT
AATTTTTTGCCATCGTTTTTTAGAGCGTGGCTTTTCATGTTGCTCACTGACCGGAGTTATCGGCACGACCATG
AAAATACCGTACAACGGGACCTCTCTCA
>P84102
CTGCATGAGTATGCCATTGCAGGGGGCAGCTTACAGATCCCTTCATGAAGTTGCAGGCTGCAGGCAAGTCATC
CTTGGCTACAGTACATGACTCTCCATTCCCATGTTGAACGTTTCCTTCCTTCCTCATAGATCAGGTAGAATTA
AATCCTC
>P88391
TAGGGTTTGTGAGGGAAAAACGACAACAGGTGAAATTGTCGCATGATCTGATCTTTCCTCAACGACGAAAACA
AGAGAGAGAAAATTTAAGATCATGCTGGCAGCTTCAACTGTTAGTAATTCACCATAATTTGAAGGGAAAAGCT
>P89332

AGGAGCCAAGTTCAGCCATGGCTGTCACGTCTTGGACTGAAGGGAGCTCCCTCGCCATCCACTACCTTGAAGG
GAAAGGAGGAACTGAGGCTCACAGATTCTGAGATCATAAAGATGCAGCAGATAAGATGAGGGCTTTTTTCC
